# The Upregulation of Toll-Like Receptor 3 via Autocrine IFN-β Signaling Drives the Senescence of Human Umbilical Cord Blood-Derived Mesenchymal Stem Cells Through JAK1

**DOI:** 10.3389/fimmu.2019.01659

**Published:** 2019-07-23

**Authors:** Hyang Ju Lee, Bongkun Choi, Yongsub Kim, Sang Eun Lee, Hye Jin Jin, Hee-Seop Lee, Eun-Ju Chang, Seong Who Kim

**Affiliations:** ^1^Department of Biochemistry and Molecular Biology, Asan Medical Center, University of Ulsan College of Medicine, Seoul, South Korea; ^2^Stem Cell Immunomodulation Research Center, Asan Medical Center, University of Ulsan College of Medicine, Seoul, South Korea; ^3^Department of Biomedical Sciences, Asan Medical Center, University of Ulsan College of Medicine, Seoul, South Korea; ^4^Biomedical Research Institute, MEDIPOST Co., Ltd., Seongnam-si, South Korea

**Keywords:** senescence, mesenchymal stromal cell (MSC), Toll-like receptor 3 (TLR3), Janus kinase 1 (JAK1), interferon-β (IFN-β)

## Abstract

Although mesenchymal stromal cells (MSCs) are among the most promising cell sources for cell-based therapies and regenerative medicine, the decline in their function with age due to cellular senescence limits their therapeutic applications. Unveiling the underlying mechanism of MSC senescence is therefore of substantial interest with regard to advancing MSC-based cell therapies. We here show that the induction of human umbilical cord blood-derived MSC (UCB-MSC) senescence causes the predominant upregulation of Toll-like receptor 3 (TLR3). Subsequent TLR3 activation by polyinosinic-polycytidylic acid triggers the prominent features of senescence. Using a clustered regularly interspaced short palindromic repeats/Cas9 library screening system, we identified Janus kinase 1 (JAK1) as the candidate regulatory factor for TLR3-mediated MSC senescence. A JAK1 deficiency blocked the MSC senescence phenotype upon TLR3 activation and TLR3 induction. Targeting the JAK1 pathway using chemical JAK1 inhibitors also significantly suppressed TLR3-mediated MSC senescence. Importantly, we further observed that UCB-MSC senescence is driven by a senescence-associated secretory phenotype (SASP) and that interferon-β (IFN-β) is a component of TLR3-dependent SASP, whereby its autocrine actions upregulate TLR3 and suppress cell proliferation. A JAK1 depletion significantly interrupted these effects of IFN-β, indicating that JAK1 is a signaling mediator linking IFN-β activity to TLR3 expression and the process of MSC senescence. Collectively, our findings provide new mechanistic insights into UCB-MSC senescence by revealing the role of an autocrine regulatory loop of SASP evoked by TLR3 activation.

## Introduction

Mesenchymal stromal cells (MSCs) are multipotent stem cells with the capacity for self-renewal and the potential to differentiate into diverse cell types, including osteoblasts, adipocytes, myocytes, neurons, hepatocytes, and chondrocytes (1). MSCs also secrete various cytokines and growth factors to regulate their various cellular functions (2). MSCs isolated from umbilical cord blood, bone marrow, and adipose tissue have been extensively investigated as a basis for cellular therapy in disease settings (3). The human umbilical cord blood-derived MSCs (UCB-MSCs) have been considered the standard choice for cell-based therapy for various diseases and for regenerative medicine because of their ready isolation from tissues, high proliferative capacity, little or no immunogenicity, and low tumorigenicity (4, 5). To acquire sufficient numbers of cells for MSC-based clinical applications, the reliable and efficient expansion of these cells *in vitro* through long-term cultivation is required. However, similar to primary tissue cells, this expansion in culture causes cellular senescence and a consequent decline in cell function which potentially compromises the use of these cells in therapeutic applications (6).

Cellular senescence can be triggered by diverse stimuli, including telomere dysfunction, oxidative stress, genomic instability, oncogenic and metabolic insults, and epigenetic changes (7). The progression of cells toward replicative senescence is accompanied by the gradual expression increase in the transcription factor p53, cyclin dependent kinase inhibitors, p21^WAF1^ (p21), and p16^INK4a^ (p16) (8). Senescence is paralleled by an increase in specific markers including senescence-associated β galactosidase (SA-β-gal), DNA damage foci, an enlarged cell size, cell cycle arrest, and the senescence-associated secretory phenotype (SASP) (9). SASP entails the secretion of proinflammatory cytokines, chemokines, growth factors, and extracellular matrix remodeling proteases (9, 10). Given that SASP components secreted by senescent MSCs increase the complexity of paracrine communication among these cells and their physiological microenvironment (11), the cellular senescence phenotype can be sustained and amplified by the autocrine or paracrine regulatory loop of SASP (12).

SASP has been previously associated with Toll like receptor (TLR) pathways (13). The TLRs are key mediators of innate and adaptive immune responses (14). Furthermore, increasing evidence has demonstrated that TLRs are expressed in MSCs and modulate various biological functions of these cells including proliferation, differentiation, migration, and immunological behavior (15). TLRs regulate the immune responses mediated by proinflammatory cytokines and type I interferons (IFNs) (16). The two types of IFN, Type I (mainly IFN-α and IFN-β) and Type II (IFN-γ) bind to their receptors and initiate a cascade of events known as the Janus kinase (JAK)/signal transducer and activator of transcription (STAT) signaling pathway which is also involved in cellular senescence (17, 18). The increased levels of circulating inflammatory markers, including IFN-β, IFN-γ, IL-1β, IL-6, and TNF-α are associated with senescence (19). Previous studies have reported that IFN-β upregulates p53-dependent senescence in normal human fibroblasts (20) and induces cellular senescence by modulating the JAK/STAT-induced promyelocytic leukemia protein (PML) in papilloma virus-transformed keratinocytes and tumor cells (7, 17, 19). Furthermore, TLR function was found to be dysregulated in the context of aging (21), implying the involvement of TLRs in senescence.

The JAK/STAT pathway plays an important role in regulating cytokine production (22) and its inhibition suppresses SASP in preadipocytes and endothelial cells (23) and reprograms the SASP in senescent tumor cells (24). JAK1 mediates the biological functions induced by three major cytokine receptor subfamilies including class II cytokine receptors (the receptors for IFN-α/β, IFN-γ, and IL-10), cytokine receptors that utilize the γ_c_ receptor subunit (the receptors for IL-2, IL-4, IL-7, IL-9, and IL-15) and cytokine receptors utilizing the gp130 subunit (the receptors for IL-6, IL-11, LIF, OSM, CNTF, and CT-1) (25). A knockdown of JAK1 inhibits cell proliferation, invasion, and *in vivo* tumor growth and disrupts the PI3K/mTOR pathway (26). JAK1, JAK2, and STAT3 are involved in cell growth, survival, invasion, and migration in colorectal cancer cells (27) and JAK inhibitors reduce inflammation and alleviate frailty in aged mice (23). Thus, the JAK pathway is considered as a crucial factor in the senescent cells, however, their role in the senescence of MSCs remains largely unclear.

In our present study, we show for the first time that TLR3 is predominantly upregulated in senescent human UCB-MSCs and that the activation of this receptor leads to MSC senescence. Additionally, we identify JAK1 as a key regulatory factor linked to the cellular senescence of MSCs triggered by TLR3 activation through the use of clustered regularly interspaced short palindromic repeats (CRISPR)/Cas9 knockout (GeCKO) library screening of MSCs after continuous treatment with polyinosinic-polycytidylic acid (poly IC). Finally, our findings indicate that JAK1 activation mediates the IFN-β action to increase TLR3 expression, thereby reinforcing TLR3-mediated MSC senescence. These findings emphasize the distinct role of IFN-β as a component of the TLR3-dependent SASP involved in TLR3-mediated MSC senescence.

## Materials and Methods

### Cell Culture and Reagents

The isolation of human MSCs was approved by the Institutional Review Board of MEDIPOST Co., Ltd. (Seongnam, Korea) and MSCs used in this study were donated by MEDIPOST Co., Ltd. Umbilical cord blood was collected from umbilical veins after neonatal delivery with maternal informed consent. Umbilical cord blood-derived MSCs were separated as previously described (28). The cells were then plated at 2 × 10^3^ cells/cm^2^ and split at ~70% confluency every 5 days. 293T cell lines were cultured in Dulbecco's modified Eagle's medium (DMEM; GE Healthcare, Chicago, IL) containing 10% FBS and penicillin/streptomycin. All cells were maintained at 37°C in a humidified 5% CO_2_ atmosphere. Poly IC was purchased from Invivogen (San Diego, CA) and tofacitinib, ruxolitinib, and GLPG0634 were obtained from Selleckchem (Houston, TX).

### Real-Time Quantitative Polymerase Chain Reaction (qPCR)

Total RNA was isolated from MSCs using TRIzol Reagent (Thermo Fisher scientific, Waltham, MA) in accordance with the manufacturer's protocol. Total RNA aliquots (2 μg) were then reverse transcribed using a TOPscript cDNA synthesis Kit (Enzynomics, Daejeon, Korea). qPCR was performed on the CFX Connect Real-Time PCR Detection System (Bio-rad, Hercules, CA). The primers used are described in Supplementary Table 1. Gene expression was normalized to that of GAPDH, which was used as an internal control. The relative quantities of mRNA of interest were calculated using the comparative threshold cycles (2^−ΔΔCt^) method.

### Western Blotting

Western blotting was performed as previously described (28). Each membrane was blocked in 0.1% Tris buffered saline with Tween 20 (TBST) containing 5% bovine serum albumin and incubated with the indicated primary antibodies against phospho-p53, p21, phospho-pRb, TLR3, JAK1, phospho-STAT1 (Cell Signaling Technology, Danvers, MA), IL-6, p16, EZH2, CyclinD1 (Abcam, Cambridge, UK), PCNA (Biosciences, San Jose, CA), BMI1 (Active Motif, Carlsbad, CA), survivin (Genetex, Irvine, CA), and β-actin (Sigma-Aldrich). The membranes were then incubated with the corresponding peroxidase-conjugated secondary antibodies for 1 h at room temperature. The blots were developed using the Advansta Western Bright ECL HRP Substrate Kits (Advansta, San Jose, CA) and detected using a C-DiGit Blot Scanner (LI-COR, Lincoln, NE). The signal of each band was quantified using Image J densitometry software (Version 1.6, National Institutes of Health, Bethesda, MD) for densitometric evaluation. The targeted protein levels were normalized to the level of β-actin.

### Enzyme Linked Immunosorbent Assay (ELISA)

The concentrations of human IFN-β (VeriKine™ Human IFN β ELISA; PBL Assay Science, Piscataway, NJ), IL-6 (Human IL-6 ELISA MAX™ Deluxe, Biolegend, San Diego, CA), IL-7 (Human IL-7 Quantikine HS ELISA Kit; R&D systems, Minneapolis, MN), and IL-15 (Human IL-15 Quantikine ELISA Kit; R&D systems) in conditioned media from human MSCs were measured in accordance with the manufacturer's protocols. All samples were examined in triplicate for each experiment.

### Senescence Associated Beta Galactosidase Staining (SA-β-Gal Staining)

Human MSCs (2 × 10^4^ cell/well) were seeded and treated with poly IC or tofacitinib. SA-β-gal staining was performed as previously described (28).

### 5-Bromo-2-Deoxyuridine (BrdU) Cell Proliferation Assay

Cell proliferation was measured using BrdU cell proliferation ELISA (Roche Applied Science, Upper Bavaria, Germany), which quantifies the incorporation of BrdU, a thymidine analog, during DNA synthesis. Cell proliferation was calculated relative to the percentage of control cells.

### Generation of the Gene Knockout MSCs Using the CRISPR/Cas9 Technique

Oligonucleotide pairs for the target sequences (JAK1, TLR3, IFNAR1) were annealed and the fragments were then cloned into the BsmB I site of plentiCRISPR v2 plasmid (Addgene, Watertown, MA, USA). The Oligonucleotides used are described in Supplementary Table 2. Lentivirus was produced via the co-transfection of the pMD2.G (Addgene) envelope and psPAX2 (Addgene) packaging plasmids into 293T cells using Neon Transfection System (Thermo Fisher scientific). The virus-containing supernatant was collected 72 h after transfection and passed through a 0.45 μm filter to eliminate cells. MSCs were infected by virus-containing supernatant. At 72 h after infection, virus was removed and cells were selected with the 2.5 μg/ml puromycin (Sigma-Aldrich).

### CRISPR/Cas9 Knockout (GeCKO) Screening

#### GeCKO Library Lentivirus Production

Lentivirus was produced via the co-transfection of 80 μg of plentiCRISPR plasmid library (Addgene) with the 40 μg pMD2.G (Addgene) envelope and 60 μg psPAX2 (Addgene) packaging plasmids into 4 × 10^7^ 293T cells using the Neon Transfection System (Thermo Fisher scientific). Cells were pulsed twice with a voltage of 1,100 and a width of 20. The virus-containing supernatant was collected 72 h after transfection and passed through a 0.45 um filter to eliminate cells. Aliquots were stored at −80°C.

#### Cell Transduction Using the GeCKO Library

To introduce the GeCKO library viruses into MSCs, 1 × 10^6^ cells were seeded and incubated overnight. Cells were then infected with 20 ml GeCKO library lentivirus-containing supernatant. At 72 h after infection, the virus was removed, and cells were selected with 2.5 μg/ml puromycin (Sigma-Aldrich) for 48 h. Puromycin-selected GeCKO-library-MSCs were maintained for 7 days and then stored in freezing medium at −80°C until use.

#### Poly IC Resistance Screen

GeCKO library-MSCs were cultured with or without poly IC (1 μg/ml). Cells were either passaged or fresh media containing poly IC was added every 2 days. The MSCs were finally harvested at 14 days after poly IC addition at which point the screen was terminated.

#### Genomic DNA Sequencing

Genomic DNA extraction, library construction, tagging (indexing) of samples, deep sequencing using the Illumina MiSeq platform, and data processing were performed by Macrogen Inc. (Seoul, Korea).

### Statistical Analysis

**Figure 1:**
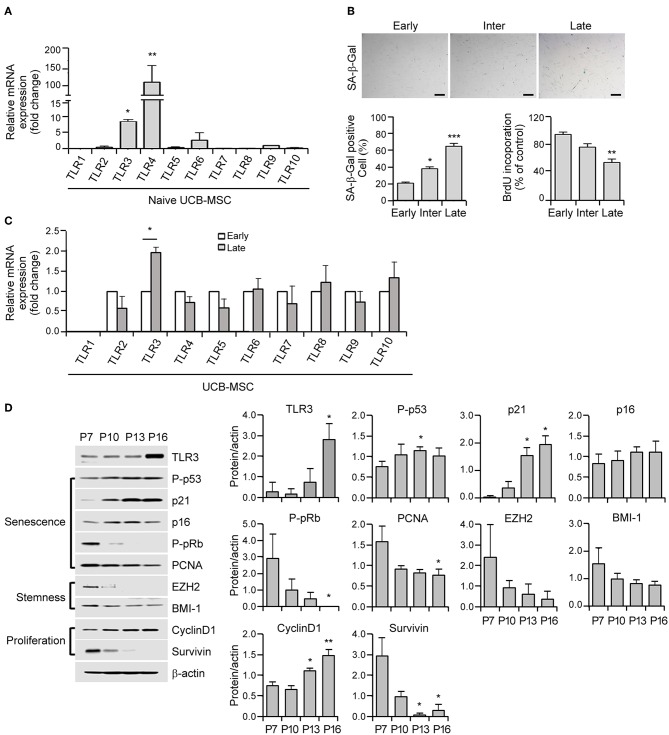
Cellular senescence leads to the upregulation of Toll-like receptor 3 (TLR3) in human umbilical cord blood-derived mesenchymal stromal cells (UCB-MSCs). **(A)** Total RNAs were isolated from human MSCs and processed for real time quantitative PCR (qPCR) analysis of TLR1–TLR10 expression. **(B)** Representative image of senescence-associated ß-galactosidase (SA-ß-gal) staining in early, intermediate, and late phase MSCs. Scale bars, 20 μm (upper panel). The percentage of SA-ß-gal-positive cells was determined. Cell proliferation in early, intermediate, and late phase MSCs was measured by 5-bromo-2-deoxyuridine (BrdU) assay (lower panel). **(C)** mRNA expression levels of TLR1–TLR10 were analyzed in early and late phase MSCs by qPCR. The transcript levels were normalized to those of GAPDH. **(D)** The protein expression levels of TLR3, markers of senescence (p-p53, p21, p16, p-pRb, and PCNA), stemness (EZH2 and BMI-1), and proliferation (cyclinD1 and survivin) factors in MSCs at the indicated passage number were determined by immunoblotting. ß-actin was used as the loading control. Densitometric quantification. Western blot was quantified and expressed as the ratio of proteins and ß-actin intensity. All data were obtained from three independent experiments. Values are the mean ± SEM (*n* = 3). *P < 0.05; **P < 0.01; ***P < 0.001. *P* values were calculated using the Kruskal-Wallis test.

**Figure 2:**
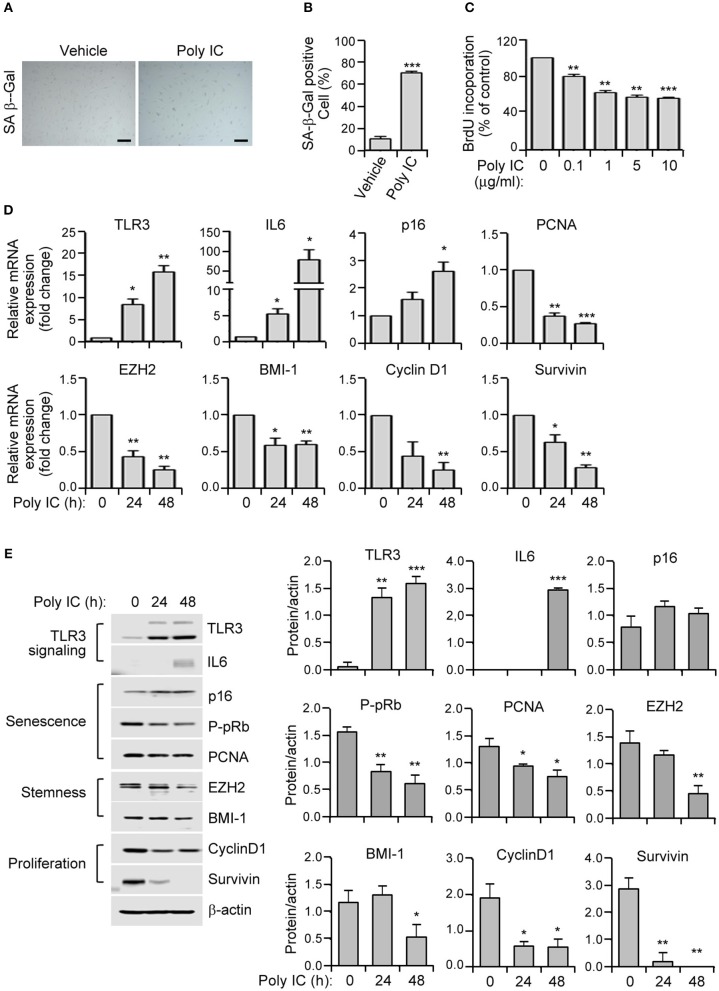
TLR3 activation triggers the prominent features of senescence in human MSCs. **(A)** SA-ß-gal staining of polyinosinic-polycytidylic acid (poly IC)-treated MSCs. Scale bars, 20 μm. **(B)** The number of SA-ß-gal positive cells was measured. **(C)** MSCs were treated with the indicated concentrations of polyinosinic-polycytidylic acid (poly IC) for 48 h and cellular proliferation was measured by BrdU incorporation. **(D)** Relative mRNA expression levels of TLR3, IL-6, p16, p-pRb, PCNA, EZH2, BMI-1, cyclinD1, and survivin were determined in MSCs treated with poly IC (1 μg/ml) at the indicated times using qPCR. These transcript levels were normalized to those of GAPDH, with the level of expression in vehicle-treated MSCs defined as 1. **(E)** The protein expression of TLR3, IL-6, and markers of senescence (p16, p-pRb, and PCNA), stemness (EZH2 and BMI-1), and proliferation (cyclinD1 and survivin) in MSCs treated with poly IC (1 μg/ml) were determined at the indicated times by immunoblotting. ß-actin was used as the loading control. Densitometric quantification. Data were obtained from three independent experiments. Values are the mean ± SEM (*n* = 3). *P < 0.05; **P < 0.01; ***P < 0.001. *P* values were calculated using the Mann-Whitney test **(B)** or Kruskal-Wallis test **(C–E)**.

All quantitative experiments were performed at least in triplicate. Data are presented as means ± standard error of the mean (SEM) of at least two independent experiments. The Mann-Whitney test (**Figures 2B**, **6A,B**) or Kruskal-Wallis test (Figures 1, 2C–E, **4**, **5**, **6B–J**) were used to determine significance. *P* < 0.05 was considered statistically significant.

## Results

### The Long-Term Expansion of Human MSCs Upregulates TLR3 Expression During Cellular Senescence

To elucidate the role of TLRs in cellular senescence in human UCB-MSCs, we analyzed their expression patterns in these cells using real time quantitative PCR (qPCR) analysis of TLR1–TLR10 mRNA. MSCs dominantly express TLR3 and TLR4 (Figure 1A). The cells were then continuously passaged at regular intervals until they lost their ability to divide. In accordance with the senescence status of the growth rate and the expression of senescence markers such as P-pRb, P-p53, and p21, MSCs were categorized into early (P1–P7), intermediate (P8–P13), and late (P14–P16) phases. Early phase MSCs showed a fast growth rate (population doubling time of early phase: 16.1 ± 4.9 h, intermediate phase: 22.8 ± 3.6 h, late phase: 43.9 ± 14.4 h) and MSCs in the intermediate stage entered early senescence as evidenced by increased SA-β-gal staining (Figure 1B). Compared with early-phase cells, late-phase MSCs displayed significantly augmented SA-β-gal positivity (Figure 1B) and severely reduced cell proliferation as revealed by decreased BrdU incorporation (Figure 1B). To examine the relationship between MSC senescence and TLR expression, we measured the expression levels of TLR1–TLR10 during replicative senescence and found that TLR3 was upregulated but other TLRs showed minimal differences under senescence conditions in human MSCs (Figure 1C).

Cellular senescence depends on two powerful molecular mechanisms: the p53 and pRb/p16 pathways. The protein expression of the p53 transcriptional target p21 shows increased expression from the intermediate phase and becomes fully activated in the late phase (Figure 1D). In contrast, phosphorylated pRb progressively decreases from the intermediate phase and becomes barely detectable in the late phase (Figure 1D). Notably, late MSCs exhibited diminished expression of stemness markers including EZH2 and BMI-1, which is associated with reduced expression of the proliferation marker, survivin (Figure 1D). Notably, actively growing MSCs displayed a low TLR3 protein level, whereas the expression of TLR3 increased as the P number increased (Figure 1D). Taken together, these results indicate that long-term cultivation of human MSCs up to the late phase induces senescence accompanied by a predominant upregulation of TLR3.

### TLR3 Activation Induces Cellular Senescence in MSCs

To investigate whether TLR3 upregulation in senescent MSC is linked to the induction of senescence, we treated MSCs in early phase of senescence with polyinosinic-polycytidylic acid (poly IC). We observed a remarkable increase in cellular senescence, as demonstrated by the induction of SA-β-gal activity by up to five-fold (Figures 2A,B), and a dose-dependent decrease in cellular proliferation as evidenced by a 5-bromo-2′-deoxyuridine (BrdU) assay (Figure 2C) compared to cells treated with a vehicle control.

We next examined the effects of TLR3 activation on the expression of senescence-related genes in MSCs. The mRNA expression of TLR3 is lower in naïve cells but became considerably upregulated at 24 h and further increased at 48 h (Figure 2D). This then increased the TLR3 protein level in a time-dependent manner (Figure 2E) after poly IC treatment, consistent with previous findings (29). Furthermore, poly IC exposure remarkably augmented the IL-6 and p16 mRNA levels (Figure 2D). This was associated with a concomitant decrease in the expression of PCNA, EZH2, BMI-1, cyclin D1, and survivin transcripts in the poly IC exposed cells, as determined by qPCR (Figure 2D). Consistently, poly IC treatment induced TLR3 and IL-6 protein expression, which was accompanied by reduced levels of stemness markers (EZH2 and BMI-1) and proliferation markers (cyclin D1 and survivin) (Figure 2E). These data indicated that poly IC-mediated TLR3 activation triggers the prominent features of senescence in MSCs.

### CRISPR/Cas9 Library Screening of TLR3 Ligand (Poly IC) Resistance Genes

**Figure 3:**
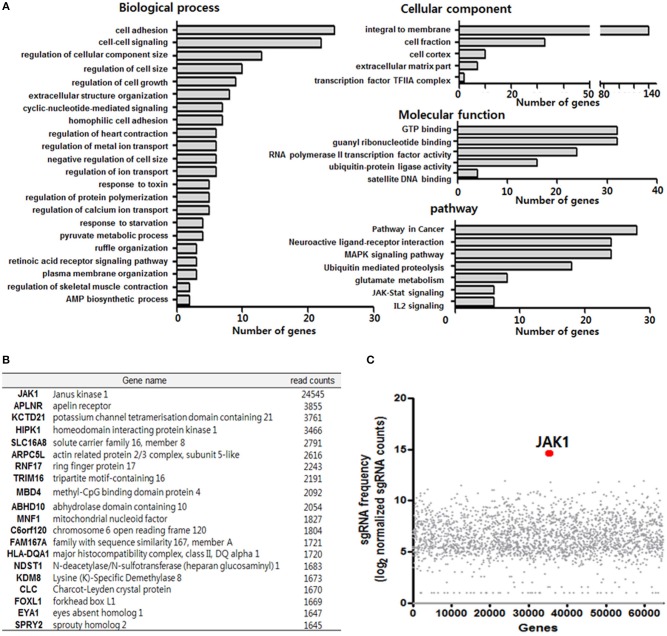
Clustered regularly interspaced short palindromic repeats (CRISPR)/Cas9 library screening of TLR3 ligand (poly IC) resistance genes. **(A)** Functional annotation clustering analysis of poly IC resistance genes was performed using the DAVID bioinformatics resource. **(B)** Gene list corresponding to the top 20 hits for resistance following TLR3 activation in MSCs. **(C)** Scatterplot showing enrichment of sgRNAs after poly IC treatment for 14 days. All data were obtained from two independent experiments.

To further determine the key regulatory factors for TLR3-mediated MSC senescence, we employed GeCKO library screening. A single lentiviral delivery vector containing Cas9, single-guide RNAs (sgRNAs), and a puromycin selection marker was used in this system. We treated the GeCKO library MSCs with poly IC (1 μg/ml) for 14 days and then harvested the cells that were resistant to continuous treatment with poly IC. We then conducted Next Generation Sequencing (NGS) analysis of the single-guide RNA (sgRNA) locus and obtained 495 candidate genes using a 500 read count cutoff value. Employing DAVID gene functional classification analysis, we observed that the genes involved in JAK-STAT, MAPK, PI3K-AKT, and ubiquitin mediated proteolysis signaling pathway were highly scored (Figure 3A). For a subset of genes, we detected the enrichment of multiple sgRNAs that target each gene after 14 days of poly IC treatment. Notably, JAK1 achieved the top score in this analysis among TLR3 ligand (poly IC) resistance genes (Figures 3B,C), indicating that it may be a key regulatory factor associated with TLR3-mediated MSC senescence.

### JAK1 Depletion Attenuates MSC Senescence Mediated by TLR3 Activation

**Figure 4:**
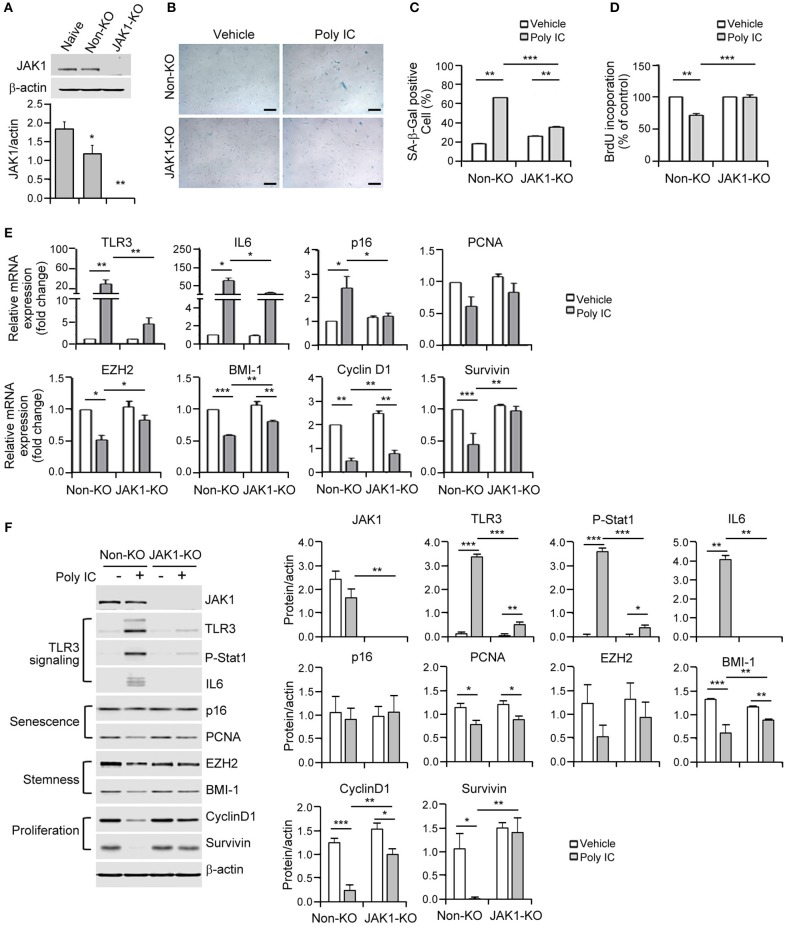
The depletion of Janus kinase 1 (JAK1) prevents MSC senescence upon TLR3 activation. **(A)** The JAK1 knockout efficiency was determined by immunoblotting of control and JAK1-knockout MSCs. **(B)** Control and JAK1-knockout MSCs were stimulated with 1 μg/ml poly IC for 48 h and stained with SA-ß-gal. Scale bars, 20 μm. **(C)** The percentage of SA-ß-gal positive cells was determined. **(D)** Cell proliferation was measured by BrdU incorporation. **(E,F)** The mRNA **(E)** and protein **(F)** expression levels of JAK1, TLR3, p-signal transducer and activator of transcription 1 (Stat1), IL-6, markers of senescence (p16 and PCNA), stemness (EZH2 and BMI-1) and proliferation (cyclinD1 and survivin) in control and JAK1 knockout MSCs treated with poly IC for 48 h. The transcript levels were normalized to those of GAPDH and ß-actin was used as the loading control. Densitometric quantification. The data were obtained from three independent experiments and the values are the mean ± SEM (*n* = 3). *P < 0.05; **P < 0.01; ***P < 0.001. *P* values were calculated using the Kruskal-Wallis test.

Given that JAK1 was found to be a potential regulator of TLR3 resistance genes, we established JAK1 knockout MSCs using a lentiviral CRISPR/Cas9 system to further elucidate its causative role in TLR3-dependent senescence. Immunoblotting analysis verified that JAK1 was fully knocked out in these cells (Figure 4A). This JAK1 depletion in MSCs significantly blocked the poly IC-induced senescent phenotype, as indicated by the reduced activation of SA-β-gal (Figures 4B,C). To quantify the rescue from senescence, we performed a BrdU-incorporation assay to measure the extent of DNA replication. The significance of JAK1 in this process was further validated by the fact that its depletion restored the poly IC-mediated decrease in cell growth (Figure 4D).

The lentiviral CRISPR/Cas9 system that successfully targeted JAK1 led to a reversal of the mRNA expression of stemness and proliferation markers including TLR3, IL-6, p16, EZH2, BMI-1, cyclin D1, and survivin following poly IC stimulation (Figure 4E). At the molecular level, JAK1 depletion abrogated the increase in TLR3 and IL-6 induced by poly IC treatment in MSCs (Figure 4F). Simultaneously, the JAK1 knockout restored the poly IC-mediated loss of cyclin D1 and survivin protein expression (Figure 4F). Previous studies have demonstrated that the JAK family members act through downstream STAT proteins to modulate various biological functions and that JAK/STAT pathway inhibition suppresses the SASP (23). Most importantly, we observed that phosphorylated STAT1 was elevated in poly IC-treated MSCs and substantially reduced by JAK1 depletion (Figure 4F). Taken together, these findings demonstrate that JAK1 depletion prevents the cellular senescence caused by TLR3 induction.

### JAK1 Inhibition Suppresses TLR3-Mediated MSC Senescence

**Figure 5:**
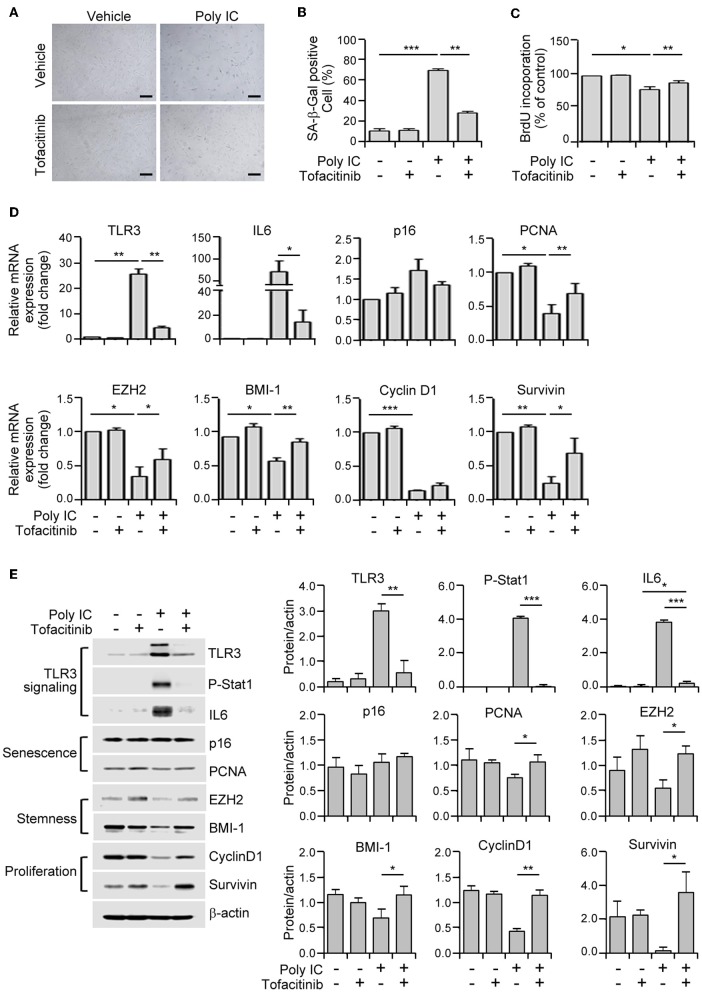
A JAK1 chemical inhibitor suppresses TLR3-mediated senescence in MSCs. **(A)** MSCs were stimulated with 1 μg/ml poly IC in the presence or absence of tofacitinib (1 μM) for 48 h and stained with SA-ß-gal. Scale bars, 20 μm. **(B)** The percentage of SA-ß-gal positive cells was determined. **(C)** Cell proliferation was measured by BrdU incorporation assay. **(D)** The relative mRNA levels of TLR3, IL-6, p16, PCNA, EZH2, BMI-1, cyclinD1, and survivin were determined by qPCR analysis of MSCs treated with poly IC and tofacitinib. The transcript levels were normalized to those of GAPDH. **(E)** The protein expression of TLR3, p-Stat1, IL-6, and of markers of senescence (p16 and PCNA), stemness (EZH2 and BMI-1), and proliferation (cyclinD1 and survivin) were determined by immunoblotting analysis of MSCs treated with poly IC in the presence or absence of tofacitinib for 48 h. ß-actin was used as the loading control. Densitometric quantification. Data were obtained from three independent experiments. The values are the mean ± SEM (*n* = 3). *P < 0.05; **P < 0.01; ***P < 0.001. *P* values were calculated using the Kruskal-Wallis test.

We further verified the role of JAK1 in TLR3-mediated senescence in MSCs through the use of tofacitinib, a known chemical inhibitor of JAK1/3 (30). The poly IC-mediated elevation of SA-β-gal positivity was reduced in these cells by exposure to this agent (Figures 5A,B). In addition, tofacitinib treatment improved the proliferative capacity of MSCs in comparison with the poly IC-treated cells (Figure 5C), thus validating the involvement of JAK1 in TLR3-mediated MSC senescence.

We then assessed the extent to which this JAK1 inhibitor affected the mRNA expression of senescence-associated genes. Tofacitinib exposure prevented the increase in the TLR3 and IL-6 mRNA levels, and the downregulation of PCNA, EZH2, BMI-1, and survivin transcripts, mediated by poly IC supplementation (Figure 5D). Consistent with this modulation of gene transcription, tofacitinib blocked the poly IC-induced protein expression of TLR3, IL-6, and phosphorylated STAT1, and the poly IC-mediated decreased protein expression of BMI-1, cyclin D1, and survivin (Figure 5E). This was further illustrated by our observation that two additional JAK1 inhibitors, ruxolitinib (JAK 1/2 inhibitor) and GLPG0634 (JAK1 inhibitor), exhibited similar effects in MSCs (Supplementary Figure 1). Taken together, our results suggest that the inhibition of JAK1 signaling suppresses cellular senescence and the loss of stemness in TLR3-activated MSCs, demonstrating the critical role of JAK1 in TLR3-mediated MSC senescence.

### IFN-β Is a Crucial Component of SASP in JAK1-Mediated MSC Senescence Through Its Enhancement of TLR3 Expression

**Figure 6:**
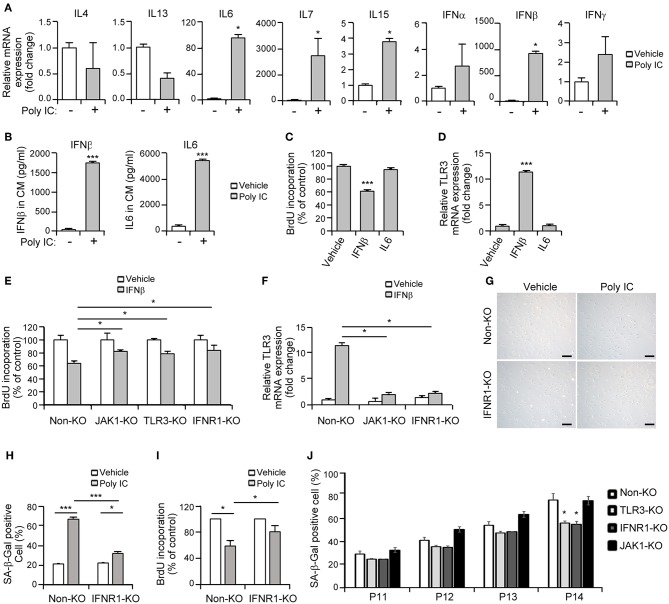
Interferon-β (IFN-β) is a crucial component of senescent-associated secretory phenotype (SASP) in TLR3-dependent MSC senescence. **(A)** mRNA expression levels of the indicated cytokines in poly IC stimulated-MSCs after 24 h were determined using qPCR. **(B)** The secreted protein levels of IFN-β and IL-6 in MSC conditioned media after poly IC treatment for 48 h were measured using ELISA. **(C)** Analysis of cell growth in MSCs treated with IFN-β (100 ng/ml) and IL-6 (100 ng/ml) for 48 h. **(D)** mRNA expression of TLR3 after treatment of MSCs with IFN-β and IL-6, as determined by qPCR. **(E,F)** Cell proliferation **(E)** and relative TLR3 mRNA expression **(F)** following INF-β treatment were measured in control, JAK1-knockout (KO), TLR3-KO, and interferon alpha and beta receptor subunit 1 (IFNAR1)-KO MSCs. The transcript levels were normalized to those of GAPDH. **(G)** Control and IFNAR1-knockout MSCs were stimulated with 1 μg/ml poly IC for 48 h and stained with SA-ß-gal. Scale bars, 20 μm. **(H)** The percentage of SA-ß-gal positive cells was determined. **(I)** Cell proliferation was measured by BrdU incorporation. **(J)** Non-KO, TLR3-KO, IFNAR1-KO, and JAK1-KO MSCs at passage 11–14 were stained with SA-ß-gal and the percentage of SA-ß-gal positive cells was determined. All data were obtained from three independent experiments. The values are the mean ± SEM (*n* = 3). *P < 0.05; ***P < 0.001. *P* values were calculated using the Mann-Whitney test **(A)** or Kruskal-Wallis test **(B–J)**.

Senescent cells exhibit the characteristic protein secretion profile, SASP (9), which sustains this phenotype (12). MSCs also secrete various cytokines and growth factors to regulate their cellular functions (2). Previous studies have demonstrated that JAK1 modulates the expression of various cytokines including IL-4, IL-6, IL-7, IL-13, IL-15, IFN-α, IFN-β, and IFN-γ (7, 25). Hence, we hypothesized that the TLR3-dependent expression of secretory cytokines in association with the JAK1 pathway may actively regulate the senescence process in MSCs. To identify the key players involved in JAK1-dependent MSC senescence, we performed qPCR analysis of JAK1-mediated cytokines in MSCs following poly IC treatment. We found that the transcript levels of four cytokines, IL-6, IL-7, IL-15, and IFN-β, were markedly increased in response to poly IC after 24 h (Figure 6A). The protein secretion of IFN-β and IL-6 was also found to be remarkably augmented compared with the vehicle control after 48 h, as determined by ELISA (Figure 6B). However, we observed only a marginal elevation in the secretion of IL-7 and IL-15 at the picogram level (Supplementary Figure 2). In contrast, the expression of IL-4, IL-13, IFN-α, and IFN-γ was largely unaffected by poly IC treatment (Figure 6A).

**Figure 7:**
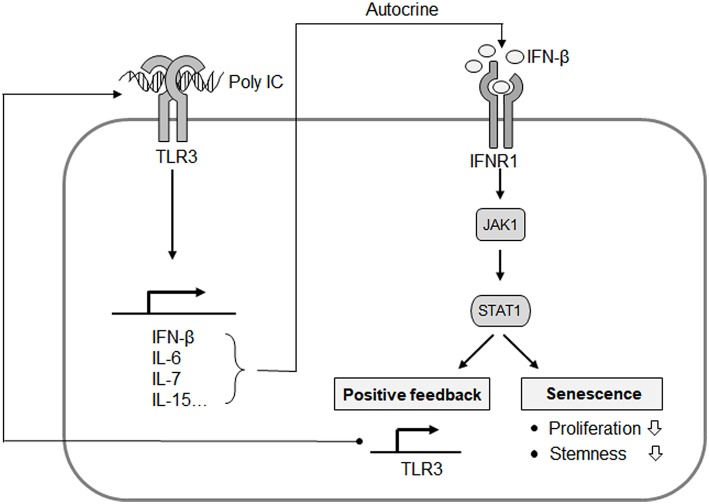
Proposed mechanistic model for the TLR3-mediated senescence of MSCs. IFN-β is a predominant component of TLR3-dependent SASP, and exerts its autocrine action to upregulate TLR3 and suppress cell proliferation. JAK1 is a signaling mediator linking IFN-β to TLR3 expression and the process of MSC senescence. This represents an autocrine regulatory loop of SASP evoked by TLR3 activation.

Thereafter, we investigated whether a cell-intrinsic senescent program could be linked to IFN-β- or IL-6-mediated SASP. A BrdU incorporation assay indicated that MSC proliferation was suppressed by IFN-β but not by IL-6 (Figure 6C). We also observed that TLR3 was upregulated in response to IFN-β treatment (Figure 6D), underlying the IFN-β-positive feedback loop that drives TLR3- and JAK1-mediated senescence. To obtain more direct evidence of the regulation of MSC senescence by IFN-β, cell proliferation was evaluated following IFN-β treatment in control, JAK1-knockout (KO), TLR3-KO, and interferon alpha and beta receptor subunit 1 (IFNAR1)-KO MSCs, respectively. The depletion of TLR3 or IFNAR1 significantly rescued the inhibitory effects of IFN-β on cell proliferation (Figure 6E), with less significant induction of TLR3 (Figure 6F). JAK1 depletion also significantly interrupted the effect of IFN-β on TLR3-mediated MSC senescence (Figures 6E,F). Furthermore, the depletion of IFNAR1 inhibited the poly IC-mediated elevation of SA-β-gal positivity (Figures 6G,H) and restored the poly IC-mediated decrease in cell growth (Figure 6I). We observed reduced number of SA-ß-gal-positive-cells in TLR3- and IFNR1-KO MSCs, however, there was no reduction in SA-ß-gal-positive-cell number in JAK1-KO MSCs at passage 14 (Figure 6J), indicating that depletion of JAK1 may be irrelevant to the induction of senescence in UCB-MSC induced by long-term *in vitro* cultivation. We concluded from this that TLR3 activation induces IFN-β secretion, which leads to MSC senescence accompanied by TLR3 upregulation. This suggests that this SASP component might be a dominant participant in TRL3-mediated MSC senescence (Figure 7).

## Discussion

Previous studies have implicated the age-dependent upregulation of human intestinal epithelial TLR3 expression (31) and TLR dysregulation in the onset of cellular senescence and the acquisition of a SASP (13). This suggested a link between TLR3 activation and senescence. Our present study has now demonstrated that TLR3 is predominantly upregulated in UCB-MSCs that become senescent through expansion in culture and that the senescent features of these cells are stimulated by TLR3 activation (Figures 1, 2). However, late-phase bone marrow-MSCs (BM-MSCs) exhibited elevated expression of TLR4 but not TLR3 accompanied with increased p16 expression (Data not shown) in consistent with a previous report (32), indicating that modulation of TLR expression by senescence may vary depending on the tissue source of the MSC. Our present findings thus verify the link between TLR3 activation and the induction of human UCB-MSC senescence.

The CRISPR/Cas9 system is an effective technique for introducing targeted mutations to specific sites in the genome (33). sgRNA, which binds to targeted regions of specific genes, directs Cas9 to induce DNA double-strand breaks, leading to frameshift insertion/deletion mutations and a loss-of-function allele (34). In our present experiments, we utilized the GeCKO library system (35) to identify key candidate regulatory factors that function in the MSC senescence mediated by TLR3 activation and identified JAK1 (Figure 3). Notably, a previous study has reported that the administration of a genotoxic drug capable of inducing senescence led to the activation of JAK1, although the authors further reported that JAK1 silencing failed to rescue senescence in human cancer cells or normal diploid fibroblasts (7). It should be emphasized however that other genes are associated with the JAK-STAT signaling pathway, including IFN, IL10Rb, STAT3, and SOCS3. IL-6 and the soluble IL-6 receptor (sIL-6R) induced premature senescence in normal human fibroblasts by establishing a senescence-inducing circuit involving STAT3 and insulin-like growth factor-binding protein 5 (36). SOCS1, which is a transcriptional target of activated STAT proteins, contributes to cellular senescence through its interaction with p53 and enhancement of the transcriptional activity of p53 (37). IL-22, which is a ligand for the receptors IL-10R2 and IL-22R1, induces the senescence of hepatic stellate cells via SOCS3-bound p53 and subsequently increases the expression of p53 and its target genes (38).

MSCs regulate their cellular functions with respect to the immune system, tissue remodeling and regeneration through the secretion of SASP components (2). Cellular senescence can be supported by secreted SASP (12). The cumulative evidence to date suggests that increased levels of circulatory cytokines, including IFN-β, IFN-γ, TNF-α, IL-1β, and IL-6, are associated with replicative senescence or premature senescence in various cell types (20, 36). Indeed, an important role of the circulatory factors in SASP, including IL-1α, IL-1β, IL-6, a number of chemokines, and matrix metalloproteinases, is highlighted by the ability of these factors to induce cellular senescence (10, 12). Since JAK/STAT signaling pathways regulate diverse development and homeostasis processes via the actions of various cytokines, hormones, and growth factors (22), it is plausible that the TLR3 activation-induced autocrine SASP plays a crucial role in the TLR3-mediated MSC senescence that may be dependent on JAK1 activation.

Previous studies have implicated that JAK2/STAT3 pathways mediate the senescence in bone marrow-derived MSCs (18), further indicating the involvement of the JAK pathway in cellular senescence. In our present study, we identified JAK1 as a key candidate regulatory factor for TLR3-mediated senescence in MSCs (Figure 3) and clearly observed that targeting JAK1 using chemical inhibitors in MSCs significantly suppresses TLR3-mediated senescence in these cells (Figure 5). In parallel with these observations, JAK1 depletion via the CRISPR/Cas9 lentiviral system caused MSCs to bypass cellular senescence, which correlated with a reversal of the suppression of stemness and proliferation markers (Figure 4). Notably, we observed in senescent MSCs that IFN-β is secreted as a dominant component of TLR3-mediated SASP, which requires JAK1 activation (Figure 6). We previously reported that monocyte chemoattractant protein-1 is also secreted as a major component of the SASP during MSC expansion and reinforces senescence via chemokine (c-c motif) receptor 2 by activating the ROS-p38MAPK-p53/p21 signaling cascade (28). It should be emphasized in this regard that the administration of IFN-β leads to increased cellular senescence in keratinocytes through the regulation of JAK/STAT-induced PML and p53, enhances p53-dependent senescence in fibroblasts, and also induces genotoxic drug-mediated senescence through JAK/STAT-mediated PML induction in tumor cells (7, 17, 19, 20). Given that IFN-β appears to reinforce MSC senescence by inducing TLR3 expression and acts in concert with its receptor through JAK1 (Figure 6), we contend that IFN-β acts in an autocrine fashion to enhance TLR3-mediated MSC senescence and that JAK1 is a signaling mediator linking the IFN-β action to TLR3-mediated MSC senescence (Figure 7).

To our knowledge, our present study provides the first clear evidence that JAK1 is a key regulatory factor in TLR3-mediated cellular senescence in human UCB-MSCs. Prior observations have demonstrated that JAK inhibitors alleviated SASP and enhance physical functions in old mice (23) and a human trial using ruxolitinib for myelofibrosis has suggested that JAK inhibitors increase human activity and reduce frailty-like symptoms (39, 40). Thus, it is plausible that JAK1 may potentiate cellular senescence through its involvement in SASP production and also act as an intracellular signaling mediator of autocrine/paracrine SASP-derived intracellular signaling activation in MSCs. The evidence now suggests that JAK1 could be an important player in poly IC-induced senescence, but not in senescence induced by long-term *in vitro* cultivation as this could modulate the positive regulatory signaling loop of TLR3-mediated cellular senescence. Instead of JAK1, TLR3 and IFNR1 may be promising targets for delaying cellular senescence in UCB-MSC. This in turn may contribute to the development of effective next-generation MSC-based cell therapies.

## Data Availability

All datasets generated for this study are included in the manuscript and/or the Supplementary Files.

## Ethics Statement

The isolation of human MSCs was approved by the Institutional Review Board of MEDIPOST Co., Ltd. (Seongnam, Korea) and MSCs used in this study were donated by MEDIPOST Co., Ltd. Umbilical cord blood was collected from umbilical veins after neonatal delivery with maternal informed consent.

## Author Contributions

HL, BC, YK, E-JC, and SK contributed to the study conception and design. HL, BC, SL, H-SL, and HJ performed the experiments and carried out the analysis and interpretation of data. HL, BC, E-JC, and SK drafted the manuscript. All authors approved the final version of the manuscript and participated in the revision of the manuscript.

### Conflict of Interest Statement

HJ was employed by company MEDIPOST Co., Ltd. The remaining authors declare that the research was conducted in the absence of any commercial or financial relationships that could be construed as a potential conflict of interest.

## Abbreviations

BrdU: 5-bromo-2′-deoxyuridine
CRISPR: clustered regularly interspaced short palindromic repeats
GeCKO: CRISPR/Cas9 knockout
IFN: interferon
IFNAR1: interferon alpha and beta receptor subunit 1
JAK: Janus kinase
KO: knockout
MSC: mesenchymal stromal cell
PML: promyelocytic leukemia protein
poly IC: polyinosinic-polycytidylic acid
qPCR: real time quantitative PCR
SASP: senescent-associated secretory phenotype
SA-β-gal: senescence-associated β galactosidase
sgRNA: Single-guide RNA
STAT: signal transducer and activator of transcription
TLR: Toll like receptor
UCB-MSC: umbilical cord blood-derived MSC.

## References

[1] De Bari C Dell'Accio F Tylzanowski P Luyten FP. Multipotent mesenchymal stem cells from adult human synovial membrane. Arthritis Rheum. (2001) 44:1928–42. 10.1002/1529-0131(200108)44:8<1928::AID-ART331>3.0.CO;2-P 11508446

[2] Hodgkinson CP Naidoo V Patti KG Gomez JA Schmeckpeper J Zhang Z. Abi3bp is a multifunctional autocrine/paracrine factor that regulates mesenchymal stem cell biology. Stem Cells. (2013) 31:1669–82. 10.1002/stem.1416 23666637 PMC3775980

[3] Glenn JD Whartenby KA. Mesenchymal stem cells: emerging mechanisms of immunomodulation and therapy. World J Stem Cells. (2014) 6:526–39. 10.4252/wjsc.v6.i5.526 25426250 PMC4178253

[4] Damien P Allan DS. Regenerative therapy and immune modulation using umbilical cord blood-derived cells. Biol Blood Marrow Transpl. (2015) 21:1545–54. 10.1016/j.bbmt.2015.05.022 26079441

[5] Jaing TH. Umbilical cord blood: a trustworthy source of multipotent stem cells for regenerative medicine. Cell Transpl. (2014) 23:493–6. 10.3727/096368914X678300 24816446

[6] Wagner W Horn P Castoldi M Diehlmann A Bork S Saffrich R. Replicative senescence of mesenchymal stem cells: a continuous and organized process. PLoS ONE. (2008) 3:e2213. 10.1371/journal.pone.0002213 18493317 PMC2374903

[7] Novakova Z Hubackova S Kosar M Janderova-Rossmeislova L Dobrovolna J Vasicova P. Cytokine expression and signaling in drug-induced cellular senescence. Oncogene. (2010) 29:273–84. 10.1038/onc.2009.318 19802007

[8] Bringold F Serrano M. Tumor suppressors and oncogenes in cellular senescence. Exp Gerontol. (2000) 35:317–29. 10.1016/S0531-5565(00)00083-8 10832053

[9] Freund A Orjalo AV Desprez PY Campisi J. Inflammatory networks during cellular senescence: causes and consequences. Trends Mol Med. (2010) 16:238–46. 10.1016/j.molmed.2010.03.003 20444648 PMC2879478

[10] Coppe JP Patil CK Rodier F Sun Y Munoz DP Goldstein J. Senescence-associated secretory phenotypes reveal cell-nonautonomous functions of oncogenic RAS and the p53 tumor suppressor. PLoS Biol. (2008) 6:2853–68. 10.1371/journal.pbio.0060301 19053174 PMC2592359

[11] Turinetto V Vitale E Giachino C. Senescence in human mesenchymal stem cells: functional changes and implications in stem cell-based therapy. Int J Mol Sci. (2016) 17:E1164. 10.3390/ijms17071164 27447618 PMC4964536

[12] Kuilman T Michaloglou C Vredeveld LC Douma S van Doorn R Desmet CJ. Oncogene-induced senescence relayed by an interleukin-dependent inflammatory network. Cell. (2008) 133:1019–31. 10.1016/j.cell.2008.03.039 18555778

[13] Olivieri F Lazzarini R Recchioni R Marcheselli F Rippo MR Di Nuzzo S. MiR-146a as marker of senescence-associated pro-inflammatory status in cells involved in vascular remodelling. Age. (2013) 35:1157–72. 10.1007/s11357-012-9440-8 22692818 PMC3705128

[14] Akira S Uematsu S Takeuchi O. Pathogen recognition and innate immunity. Cell. (2006) 124:783–801. 10.1016/j.cell.2006.02.015 16497588

[15] Wang ZJ Zhang FM Wang LS Yao YW Zhao Q Gao X. Lipopolysaccharides can protect mesenchymal stem cells (MSCs) from oxidative stress-induced apoptosis and enhance proliferation of MSCs via Toll-like receptor(TLR)-4 and PI3K/Akt. Cell Biol Int. (2009) 33:665–74. 10.1016/j.cellbi.2009.03.006 19376254

[16] van Duin D Medzhitov R Shaw AC. Triggering TLR signaling in vaccination. Trends Immunol. (2006) 27:49–55. 10.1016/j.it.2005.11.005 16310411

[17] Hubackova S Novakova Z Krejcikova K Kosar M Dobrovolna J Duskova P. Regulation of the PML tumor suppressor in drug-induced senescence of human normal and cancer cells by JAK/STAT-mediated signaling. Cell Cycle. (2010) 9:3085–99. 10.4161/cc.9.15.12521 20699642

[18] Ji J Wu Y Meng Y Zhang L Feng G Xia Y. JAK-STAT signaling mediates the senescence of bone marrow-mesenchymal stem cells from systemic lupus erythematosus patients. Acta Biochim Biophys Sinica. (2017) 49:208–15. 10.1093/abbs/gmw134 28177455

[19] Chiantore MV Vannucchi S Accardi R Tommasino M Percario ZA Vaccari G. Interferon-beta induces cellular senescence in cutaneous human papilloma virus-transformed human keratinocytes by affecting p53 transactivating activity. PLoS ONE. (2012) 7:e36909. 10.1371/journal.pone.0036909 22615843 PMC3353995

[20] Moiseeva O Mallette FA Mukhopadhyay UK Moores A Ferbeyre G. DNA damage signaling and p53-dependent senescence after prolonged beta-interferon stimulation. Mol Biol Cell. (2006) 17:1583–92. 10.1091/mbc.e05-09-0858 16436515 PMC1415317

[21] Shaw AC Panda A Joshi SR Qian F Allore HG Montgomery RR. Dysregulation of human Toll-like receptor function in aging. Ageing Res Rev. (2011) 10:346–53. 10.1016/j.arr.2010.10.007 21074638 PMC3633557

[22] Yu H Pardoll D Jove R. STATs in cancer inflammation and immunity: a leading role for STAT3. Nat Rev Cancer. (2009) 9:798–809. 10.1038/nrc2734 19851315 PMC4856025

[23] Xu M Tchkonia T Ding H Ogrodnik M Lubbers ER Pirtskhalava T. JAK inhibition alleviates the cellular senescence-associated secretory phenotype and frailty in old age. Proc Natl Acad Sci USA. (2015) 112:E6301–10. 10.1073/pnas.1515386112 26578790 PMC4655580

[24] Toso A Revandkar A Di Mitri D Guccini I Proietti M Sarti M. Enhancing chemotherapy efficacy in Pten-deficient prostate tumors by activating the senescence-associated antitumor immunity. Cell Rep. (2014) 9:75–89. 10.1016/j.celrep.2014.08.044 25263564

[25] Rodig SJ Meraz MA White JM Lampe PA Riley JK Arthur CD. Disruption of the Jak1 gene demonstrates obligatory and nonredundant roles of the Jaks in cytokine-induced biologic responses. Cell. (1998) 93:373–83. 10.1016/S0092-8674(00)81166-6 9590172

[26] Liu D Huang Y Zeng J Chen B Huang N Guo N. Down-regulation of JAK1 by RNA interference inhibits growth of the lung cancer cell line A549 and interferes with the PI3K/mTOR pathway. J Cancer Res Clin Oncol. (2011) 137:1629–40. 10.1007/s00432-011-1037-6 21861134 PMC11828260

[27] Xiong H Zhang ZG Tian XQ Sun DF Liang QC Zhang YJ. Inhibition of JAK1, 2/STAT3 signaling induces apoptosis, cell cycle arrest, and reduces tumor cell invasion in colorectal cancer cells. Neoplasia. (2008) 10:287–97. 10.1593/neo.07971 18320073 PMC2259457

[28] Jin JH Lee HJ Heo J Lim J Kim M Kim MK. Senescence associated MCP-1 secretion is dependent on a decline in BMI1 in human mesenchymal stromal cells. Antioxid Redox Signal. (2015) 24:471–85. 10.1089/ars.2015.6359 26573462 PMC4800271

[29] Mastri M Shah Z McLaughlin T Greene CJ Baum L Suzuki G. Activation of Toll-like receptor 3 amplifies mesenchymal stem cell trophic factors and enhances therapeutic potency. Am J Physiol Cell Physiol. (2012) 303:C1021–33. 10.1152/ajpcell.00191.2012 22843797 PMC3492833

[30] Hsu L Armstrong AW. JAK inhibitors: treatment efficacy and safety profile in patients with psoriasis. J Immunol Res. (2014) 2014:283617. 10.1155/2014/283617 24883332 PMC4027021

[31] Pott J Stockinger S Torow N Smoczek A Lindner C McInerney G. Age-dependent TLR3 expression of the intestinal epithelium contributes to rotavirus susceptibility. PLoS Pathog. (2012) 8:e1002670. 10.1371/journal.ppat.1002670 22570612 PMC3343008

[32] Fafian-Labora J Lesende-Rodriguez I Fernandez-Pernas P Sangiao-Alvarellos S Monserrat L Arntz OJ Effect of age on pro-inflammatory miRNAs contained in mesenchymal stem cell-derived extracellular vesicles. Sci Rep. (2017) 7:43923 10.1038/srep43923 28262816 PMC5338265

[33] Mali P Yang L Esvelt KM Aach J Guell M DiCarlo JE. RNA-guided human genome engineering via Cas9. Science. (2013) 339:823–6. 10.1126/science.1232033 23287722 PMC3712628

[34] Jinek M Chylinski K Fonfara I Hauer M Doudna JA Charpentier E. A programmable dual-RNA-guided DNA endonuclease in adaptive bacterial immunity. Science. (2012) 337:816–21. 10.1126/science.1225829 22745249 PMC6286148

[35] Shalem O Sanjana NE Hartenian E Shi X Scott DA Mikkelsen TS. Genome-scale CRISPR-Cas9 knockout screening in human cells. Science. (2014) 343:84–7. 10.1126/science.1247005 24336571 PMC4089965

[36] Kojima H Kunimoto H Inoue T Nakajima K. The STAT3-IGFBP5 axis is critical for IL-6/gp130-induced premature senescence in human fibroblasts. Cell Cycle. (2012) 11:730–9. 10.4161/cc.11.4.19172 22374671

[37] Mallette FA Calabrese V Ilangumaran S Ferbeyre G. SOCS1, a novel interaction partner of p53 controlling oncogene-induced senescence. Aging. (2010) 2:445–52. 10.18632/aging.100163 20622265 PMC2933891

[38] Kong X Feng D Wang H Hong F Bertola A Wang FS. Interleukin-22 induces hepatic stellate cell senescence and restricts liver fibrosis in mice. Hepatology. (2012) 56:1150–9. 10.1002/hep.25744 22473749 PMC3394879

[39] Tefferi A Litzow MR Pardanani A. Long-term outcome of treatment with ruxolitinib in myelofibrosis. N Engl J Med. (2011) 365:1455–7. 10.1056/NEJMc1109555 21995409

[40] Verstovsek S Mesa RA Gotlib J Levy RS Gupta V DiPersio JF. A double-blind, placebo-controlled trial of ruxolitinib for myelofibrosis. N Engl J Med. (2012) 366:799–807. 10.1056/NEJMoa1110557 22375971 PMC4822164

