# Supplementary material for: The Upregulation of Toll-Like Receptor 3 via Autocrine IFN-β Signaling Drives the Senescence of Human Umbilical Cord Blood-Derived Mesenchymal Stem Cells Through JAK1
Source: Front Immunol. 2019 Jul 23;10:1659. doi: 10.3389/fimmu.2019.01659 (PMC6665952; doi:10.3389/fimmu.2019.01659)
Supplement: Supplementary file 1 [file Data_Sheet_1.docx]

**Supplementary information**


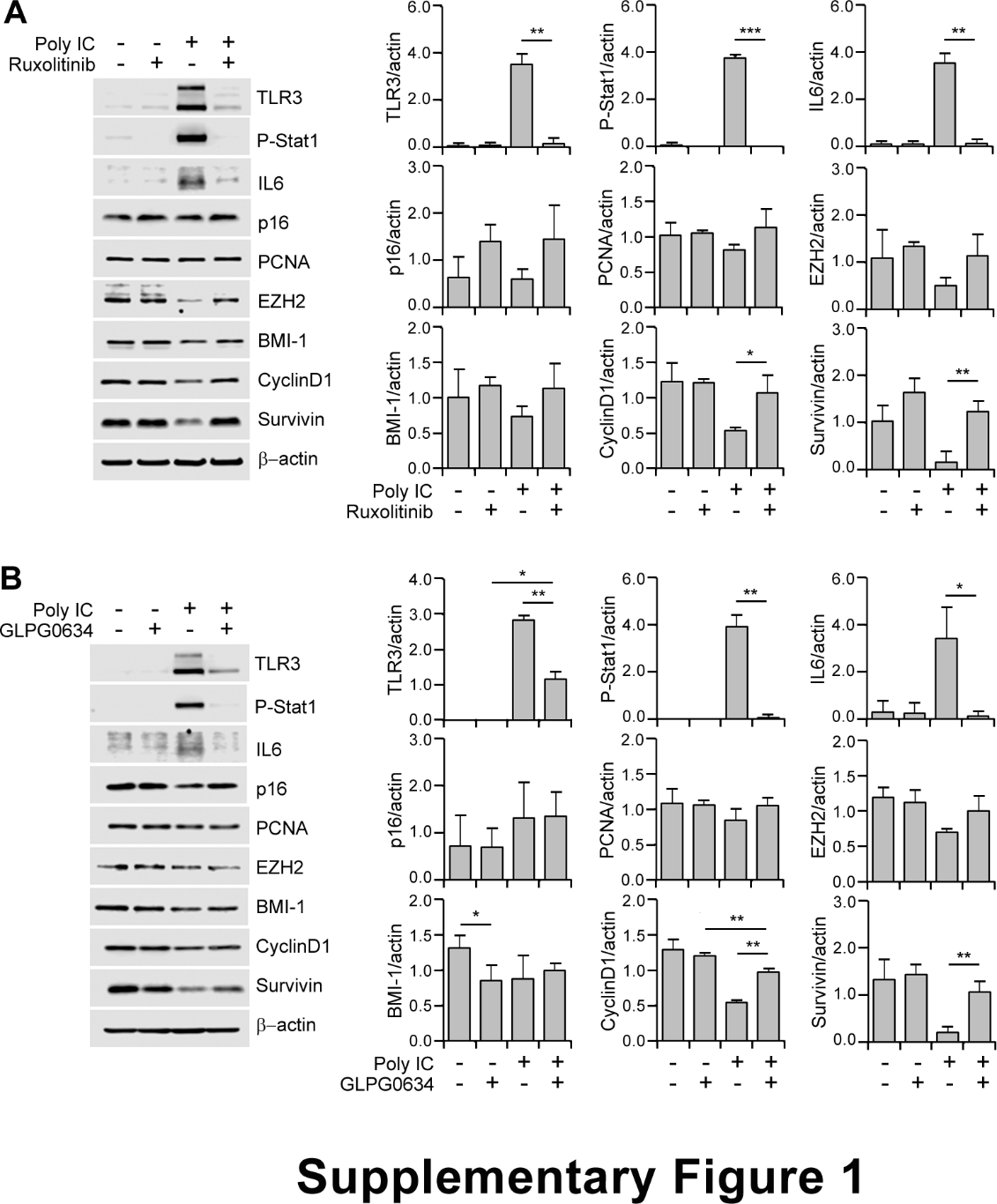


**Supplementary FIGURE 1.** Inhibition of TLR3-mediated senescence by a JAK1 inhibitor in umibilical cord blood-derived MSC (UCB-MSC). (**A**, **B**) The protein expression of TLR3 signaling (TLR3 and IL-6), senescence (p16, p-pRb, and PCNA), stemness (EZH2 and BMI-1), and proliferation (cyclinD1 and survivin) markers in MSCs treated with poly IC in the presence or absence of ruxolitinib (**A**) or GLPG0634 (**B**) for 48 hours was determined by immunoblotting. Densitometric quantification of panel. Western blot was quantified and expressed as the ratio of proteins and ß-actin intensity. All data were obtained from three independent experiments.


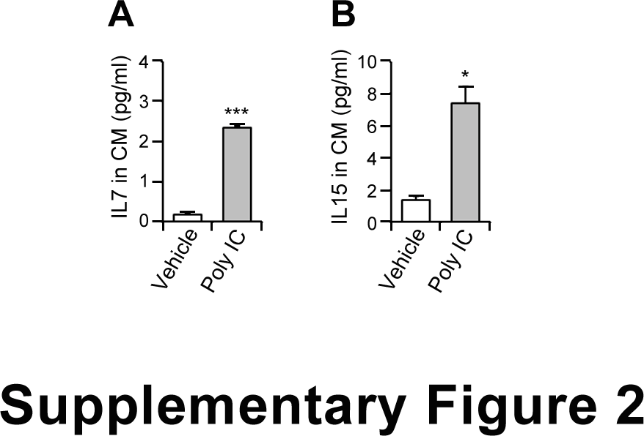


**Supplementary FIGURE 2.** Modest induction of IL-7 and IL-15 by poly IC. (**A**, **B**) The secreted protein levels of IL-7 (**A**) and IL-15 (**B**) in conditioned UCB-MSC media after poly IC treatment were measured using ELISA. All data were obtained from two independent experiments. Values are the mean ± SEM (n=3). *P < 0.05; ***P< 0.001. P values were calculated using the Mann-Whitney test.

Supplementary Table 1. Primer sequences for qPCR.

| **Target gene** | **Primer sequence (5'→3')** | |
| --- | --- | --- |
| TLR1 | Forward | TTGGAGTTCTTCTAAGGGTATGTTCC |
|  | Reverse | CTGGTATCTCAGGATGGTGTGC |
| TLR2 | Forward | GACACCAGTGCTGTCCTGTGAC |
|  | Reverse | CAAAGTCTTGATTGATTGGCCAG |
| TLR3 | Forward | TGGTGAAGGAGAGCTATCCACA |
|  | Reverse | TCCCAAGCCTTCAACGACTG |
| TLR4 | Forward | CTGAGCAGGGTCTTCTCCAC |
|  | Reverse | AAGCCGAAAGGTGATTGTTG |
| TLR5 | Forward | CACTGAGACTCTGCTATACAAGCTA |
|  | Reverse | TCGAGCCCCTACAAGGGAA |
| TLR6 | Forward | ACCTGAAGCTCAGCGATGTAGTTC |
|  | Reverse | CTATTGTTAAAAGCTTCCATTTTGT |
| TLR7 | Forward | TCAAGGCTGAGAAGCTGTAAGCTA |
|  | Reverse | TTACCTGGATGGAAACCAGCTAC |
| TLR8 | Forward | TGTCGATGATGGCCAATCC |
|  | Reverse | GAGAGCCGAGACAAAAACGTTC |
| TLR9 | Forward | CACTCGGAGGTTTCCCAGC |
|  | Reverse | TGGTGTTGAAGGACAGTTCTCTC |
| TLR10 | Forward | TGGAGTTGAAAAAGGAGGTTATAG |
|  | Reverse | GAAAGGTTCCCGCAGACTTG |
| IL-6 | Forward | CAGCTCTGGCTTGTTCCTCAC |
|  | Reverse | CAATGAGGAGACTTGCCTGGTG |
| p16 | Forward | GGGTCGGGTAGAGGAGGTG |
|  | Reverse | GCCTCCGACCGTAACTATTCG |
| PCNA | Forward | GGACATACTGGTGAGGTTCAC |
|  | Reverse | CACGTCTCTTTGGTGCAGCTC |
| EZH2 | Forward | GGACCACAGTGTTACCAGCAT |
|  | Reverse | GTGGGGTCTTTATCCGCTCAG |
| BMI-1 | Forward | TTCTTTGACCAGAACAGATTGG |
|  | Reverse | GCATCACAGTCATTGCTGCT |
| CyclinD1 | Forward | TGGAGCCCGTGAAAAAGAGC |
|  | Reverse | TCTCCTTCATCTTAGAGGCCAC |
| Survivin | Forward | CCGGACGAATGCTTTTTATG |
|  | Reverse | GCCCAGTGTTTCTTCTGCTT |
| IL4 | Forward | TCTTTGCTGCCTCCAAGAACA |
|  | Reverse | GTAGAACTGCCGGAGCACAG |
| IL7 | Forward | TTCCTCCCCTGATCCTTGTTC |
|  | Reverse | CTTGCGAGCAGCACGGAATA |
| IL13 | Forward | TTTGTTGACCACGGTCATTGC |
|  | Reverse | GAGCCTTCTGGTTCTGGGTG |
| IL15 | Forward | TTTCAGTGCAGGGCTTCCTAA |
|  | Reverse | GGGTGAACATCACTTTCCGTAT |
| IFNα | Forward | CAACCAGTTCCAAAAGGCTGAAAC |
|  | Reverse | CATCAGGGGAGTCTCTGTCAC |
| IFNβ | Forward | AGGGGAAAACGCATGAGCAG |
|  | Reverse | TGGCCTTCAGGTAATGCAGA |
| IFNγ | Forward | GAGTGTGGAGACCATCAAGGA |
|  | Reverse | TTAGCTGCTGGCGACAGTTC |
| GAPDH | Forward | TGAACGGGAAGCTCACTGG |
|  | Reverse | TCCACCACCCTGTTGCTGTA |

Supplementary Table 2. DNA oligonucleotides for vector construction.

| **Target gene** | **Primer sequence (5'→3')** | |
| --- | --- | --- |
| Non-ko | Forward | CACCGACGGAGGCTAAGCGTCGCAA |
|  | Reverse | AAACTTGCGACGCTTAGCCTCCGTC |
| JAK1 | Forward | CACCGATGACGAGAACACCAAGCTC |
|  | Reverse | AAACGAGCTTGGTGTTCTCGTCATC |
| TLR3 | Forward | CACCGTTCAACGACTGATGCTCCGA |
|  | Reverse | AAACTCGGAGCATCAGTCGTTGAAC |
| IFNAR1 | Forward | CACCGTAGATGACAACTTTATCCTG |
|  | Reverse | AAACCAGGATAAAGTTGTCATCTAC |
